# Engineering Antigens to Assemble into Polymer Particle Vaccines for Prevention of *Streptococcus suis* Infection

**DOI:** 10.3390/vaccines9121386

**Published:** 2021-11-24

**Authors:** Zennia Jean C. Gonzaga, Shuxiong Chen, Mélanie Lehoux, Mariela Segura, Bernd H. A. Rehm

**Affiliations:** 1 Centre for Cell Factories and Biopolymers (CCFB), Griffith Institute for Drug Discovery, Griffith University, Don Young Road, Natha, QLD 4111, Australia; jean.gonzaga@griffithuni.edu.au (Z.J.C.G.); shuxiong.chen@griffith.edu.au (S.C.); 2 Research Group on Infectious Diseases in Production Animals and Swine and Poultry Infectious Diseases Research Centre, Faculty of Veterinary Medicine, Université de Montréal, 3200 Rue Sicotte, CP5000, St-Hyacinthe, QC J2S 7C6, Canada; melanie.lehoux@umontreal.ca (M.L.); mariela.segura@umontreal.ca (M.S.); 3 Menzies Health Institute Queensland (MHIQ), Griffith University, Gold Coast, QLD 4222, Australia

**Keywords:** *Streptococcus suis*, particulate vaccine, biopolymer, subunit vaccine

## Abstract

*Streptococcus suis* is a zoonotic pathogen affecting pigs and humans. This bacterium causes severe economic losses in the swine industry and poses a serious threat to public health and food safety. There is no effective commercial vaccine available for pigs or humans. In this study, we applied the biopolymer particle (BP) vaccine technology to incorporate seven conserved *S. suis* antigens (38 kDa protein (38), enolase (Enol), SSU1915, SSU1355, SSU0185, SSU1215, and SSU1773 (SSU1 and SSU2)). Two combinations of these antigens (38 and Enol; all SSU antigens designated as SSU1 and SSU2) were engineered to mediate production of BPs coated with either antigens 38 and Enol or SSU1 and SSU2 inside recombinant *Escherichia coli*. The isolated and purified empty BPs, 38-BP-Enol and SSU1-BP-SSU2, showed size ranges of 312–428 nm and 292–344 nm with and without the QuilA^®^ adjuvant, respectively, and all showed a negative surface charge. Further characterization of purified BPs confirmed the presence of the expected antigen-comprising fusion proteins as assessed by tryptic peptide fingerprinting analysis using quadrupole time-of-flight mass spectrometry and immunoblotting. Vaccination with 38-BP-Enol and SSU1-BP-SSU2 formulated with and without QuilA^®^ adjuvant induced significant antigen-specific humoral immune responses in mice. Antigen-coated BPs induced significant and specific Ig (IgM + IgG) and IgG immune responses (1.0 × 10^6^–1.0 × 10^7^) when compared with mice vaccinated with empty BPs. Functionality of the immune response was confirmed in challenge experiments using an acute murine *S. suis* infection model, which showed 100% survival of the 38-BP-Enol and SSU1-BP-SSU2 vaccinated mice compared to 70% survival when vaccinated with empty BPs. Overall, our data suggest that *S. suis* antigen-coated BPs could be developed into particulate vaccines that induce protective immunity against *S. suis* infections.

## 1. Introduction

*Streptococcus suis* is a Gram-positive bacterium and the major bacterial pathogen in pigs leading to significant economic losses to the swine industry worldwide. The natural habitat of *S. suis* in pigs is the upper respiratory tract, predominantly the tonsils and nasal cavities, as well as genital and alimentary tracts [1,2]. Meningitis is the most important clinical feature linked to *S. suis* infection in pigs. In addition, arthritis, endocarditis, and septicemia with sudden death have been described. *S. suis* is a zoonotic pathogen that can be transmitted to humans. Infections in humans were considered sporadic in people exposed to contaminated pork-derived products or when in contact with the infected pigs. A significant and deadly human outbreak in China during the summer of 2005 changed the view of the threat caused by this pathogen to humans [3,4]. In humans, the infection caused by *S. suis* usually causes meningitis but also endocarditis, cellulitis, peritonitis, and arthritis [4]. Since the outbreak, the evolving zoonotic pathogen has progressively been associated with life-threatening infections relating to the streptococcal toxic shock-like syndrome [5]. *S. suis* has become the most usual cause of human meningitis in Vietnam, second in Thailand, and third in Hong Kong. In general, Asian countries are at risk of *S. suis* infections, while in Western countries it is considered as an occupational disease [3,4,5,6]. *S. suis* is undoubtedly posing a threat to public health and food safety and is considered as an emerging human pathogen [7].

Currently, antibiotic prophylaxis/metaphylaxis is the most common method to control *S. suis* infections; however, other prevention strategies are urgently needed due to increased awareness of antimicrobial resistance and, consequently, restrictions in the use of antimicrobials in livestock production. Vaccines should be a key component of strategies for preventing *S. suis* disease. However, there are currently no commercial efficacious vaccines against *S. suis* available. Autogenous vaccines (“bacterins”) are thus widely used in the field. However, field studies with autogenous vaccines or experimental studies with laboratory-made bacterins have provided contradictory results on the efficacy of this type of vaccine [1,8]

The complexity of *S. suis* epidemiology (multiple strains, serotypes, and sequence types) is making it increasingly difficult to control and manage the disease caused by these bacteria such as by the development of efficient subunit vaccines [5]. Currently, *S. suis* comprises 29 serotypes based on antigenically different structures of the capsular polysaccharide (CPS) surrounding the bacterium [9]. However, their geographical distributions differ [5,10], complicating the epidemiological portrait.

Recently, research has focused on subunit vaccines with defined antigens aiming to develop universal protective vaccines. A DNA region from the most virulent strain of *S. suis* serotype 2 encoding a 38 kDa polypeptide was identified in most *S. suis* serotypes. Pigs immunized with recombinant 38 kDa protein developed antigen-specific immune responses, resulting in complete protection against challenge with the wild-type virulent strain of *S. suis* type 2 [11]. Another promising antigen is the enolase, a multifunctional protein found at the surface of cells of most streptococcal groups and serotypes [12,13]. Enolase is highly conserved and showed protection against both serotypes 2 and 7 [14]. A more recent study identified five novel antigens (SSU1915, SSU1355, SSU0185, SSU1215, and SSU1773) through functional genomics as conserved, predicted, surface-associated proteins. They were evaluated with different adjuvant formulations in a mouse model using intranasal and intramuscular administration routes. Results showed induction of IgG antibodies to each individual protein and cellular immunity to the pool of proteins, resulting in significant protection from challenge with virulent *S. suis* [15]. In addition, the candidate vaccines induced IgG antibodies reactive against different *S. suis* serotypes, implying potential cross-protection of these subunit vaccines. While these subunit vaccine candidates show promise, their production is costly due to laborious production and purification processes as required for soluble proteinaceous antigens plus the need for adjuvants [16,17].

To overcome these disadvantages, we applied the biopolymer particle (BP) vaccine technology by engineering *E. coli* to assemble BPs displaying *S. suis* antigens to serve as particulate vaccines that induce protective immunity in pigs against *S. suis* infection. The BP core is made of polyhydroxybutyrate (PHB) that is naturally synthesized by several bacteria as carbon and energy storage [18]. BPs are made of natural polyesters comprising (*R*)-3-hydroxyfatty acid constituents and are produced and deposited in the cytoplasm as spherical, water-insoluble inclusions. The polyester resides in their core while proteins including PHB synthase (PhaC) make up the shell [19]. PhaC is the key enzyme for BP biosynthesis catalyzing the polymerization of (*R*)-3-hydroxybutyryl-CoA thioester precursors to produce BPs [18]. The BP vaccine technology has been applied for development of vaccine candidates against COVID-19 [20], *Mycobacterium tuberculosis* [21], Hepatitis C virus [22], *Neisseria meningitidis* [23], and *Streptococcus pneumoniae* [23] that all induced protective immunity in animal models. In addition, BPs showed self-adjuvanting properties, suggesting potential omission of additional adjuvants [19,23]. The use of BPs in vaccine design is an attractive alternative for *S. suis* vaccine development due to the numerous advantages of this platform including ease of manufacturing leading to low-cost production combined with strongly enhanced immunogenicity of attached antigens. In addition, the endotoxin-free *E. coli* ClearColi^TM^ BL21 (DE3) was used to manufacture the BP vaccines, avoiding laborious and costly downstream endotoxin removal steps. Importantly, the Food and Drug Administration (FDA) approved the use of the BP core polymer, PHB, for human uses [24,25]. Hence, the BP vaccine technology combined with the highly conserved antigens, 38 kDa protein (38), enolase (Enol), SSU1915, SSU1355, SSU0185, SSU1215, and SSU1773 (SSU1 and SSU2), that showed cross-protection might lead to the development of a universal vaccine that not only protects against various serotypes and strains of *S. suis* but is also applicable to the swine industry while being suitable for humans use.

## 2. Materials and Methods

### 2.1. Bacterial Strains and Growth Conditions

Table S1 shows all bacterial strains, plasmids, and primers used in this study. *E. coli* XL1-Blue strain (San Diego, CA, USA) was grown in Luria Broth (LB) medium (Thermo Fisher Scientific, Waltham, MA, USA) at 37 °C supplemented with ampicillin (Amp), 100 µg/mL) (Invitrogen, Carlsbad, CA, USA) for plasmid propagation. For BP production, *E. coli* strain ClearColi^TM^ BL21 (DE3) (Lucigen, Middleton, WI, USA), the osmosensitive variant, was grown in LB medium (Thermo Fisher Scientific) supplemented with 1% NaCl, Amp, 100 μg/mL and chloramphenicol (Cm), and 50 μg/mL (Invitrogen). *S. suis* serotype 2 strain P1/7, a well-characterized virulent reference strain [26], was grown as previously described and used for the challenge study [27]. Primers were synthesized by Integrated DNA Technologies (IDT, Coralville, IA, USA).

### 2.2. Epitope Prediction

Vaccine design for the 38-BP-Enol and SSU1-BP-SSU2 vaccines were done first by detecting antigenic epitopes of 38 kDa polypeptide, enolase, and the five novel antigens (SSU1915, SSU1355, SSU0185, SSU1215, and SSU1773). Antigenicity was screened by Jameson–Wood guide [28], surface probability by the Emni method [29], and hydrophilicity by Kyte–Doolittle [30]. The predicted epitopes were further confirmed by the freely available databases: Bepipred [31], BCPred [32], AAP, and FBCPred [33].

### 2.3. Plasmid Construction for In Vivo Production of BP Vaccines

Cloning strategies were done as previously described [34]. *E. coli* codon-optimized DNA fragments purchased from Biomatik (Kitchener, ON N2C 1N6, Canada) were used to construct two plasmids in this study (Supplementary Material, Table S1; Figure S1): (1) pET14b_38-PhaC-Enol and (2) pET14b_SSU1-PhaC-SSU2. The cloning techniques are demonstrated in Figure S1. The DNA fragments (Biomatik) were excised by enzyme digestion, and fragment separation was done using agarose gel electrophoresis with SYBR safe stain (Invitrogen). Subsequently, the target bands were excised and subjected to gel purification (Zymo Research, Irvine, CA, USA). The plasmids were isolated using High Pure Plasmid Isolation Kit (Roche, Basel, Switzerland). The final plasmid constructs were confirmed by DNA sequencing (Griffith University, Nathan, Australia). Transformation of the chemically competent *E. coli* strain ClearColi^TM^ BL21 (DE3) (Lucigen) was used to introduce the confirmed plasmids for BP production.

### 2.4. Production of BP Vaccines

*E. coli* strain ClearColi^TM^ BL21 (DE3) (Lucigen) production hosts harboring pMCS69 plasmid containing the BP precursor synthesis and the respective pET14b plasmids encoding PhaC and PhaC-fusion proteins were grown in LB media (Thermo Fisher Scientific) at 37 °C and 200 rpm as previously described [35,36]. Bioreactor vessels (Eppendorf BioFlo 320, Waterloo Rd, Macquarie Park NSW, Australia) were used and prepared according to the manufacturer’s instructions. The main cultures were prepared using animal component-free synthetic mineral media using glucose as the sole carbon source.

The cultures were grown until optical density 600 (OD_600_) reached 0.5−0.8, and fusion protein production was induced by adding 1 mM isopropyl β-D-1- thiogalactopyranoside (Sigma-Aldrich, St. Louis, MO, USA). Cells were harvested by centrifugation and mechanically disrupted, and BPs were isolated as previously described [21,22,23,37,38]. The sterile BP vaccines were stored at 4 °C in tris buffered saline (TBS, pH 7.5) until formulation for animal trials and analysis.

### 2.5. Characterization of the BP Vaccines

The whole cells and the purified BPs were characterized by transmission electron microscopy (TEM) for morphology analysis to confirm the accumulation, shape, and size of the spherical beads [35]. The purified BPs (pH 7.5) were analyzed for particle size and zeta-potential using Zetasizer Nano ZS (Malvern Panalytical) at room temperature (25 °C). The BPs 20% (*w*/*v*) were diluted to 0.1% (*w*/*v*) and particle size was evaluated by dynamic light scattering (DLS) analysis. The zeta-potential of the purified BPs was measured by electrophoretic light scattering coupled with phase analysis light scattering. Three technical replicates were performed.

### 2.6. Characterization of the Antigens/Epitopes Attached to the BPs

The antigens/epitopes coating the BPs were separated and analyzed by sodium dodecyl sulfate-polyacrylamide gel electrophoresis (SDS-PAGE) as described elsewhere [34,35,39]. The fusion protein percentage of the total protein in BP fractions was determined by densitometry using bovine serum albumin (BSA) standards (62.5 ng to 500 ng), as previously described [40]. The images were captured using gel doc (BioRad Laboratories, Hercules, CA, USA) and analyzed with the Image Lab software (BioRad Laboratories, Hercules, CA, USA). To verify the identity of the selected antigens/epitopes, Coomassie-stained protein bands of interest were excised and processed for quadrupole time-of-flight mass spectrometry (Q-TOF-MS) analysis at Clinical Research Center, The University of Queensland, Brisbane, Australia [35].

### 2.7. Ethics Statement

All experiments involving mice were carried out in accordance with the recommendations of the guidelines and policies of the Canadian Council on Animal Care and the principles set forth in the Guide for the Care and Use of Laboratory Animals. The protocols and procedures were approved by the Animal Welfare Committee of the University of Montreal (protocol number rech-1523), including euthanasia to minimize animal suffering, which was applied throughout this study when animals were seriously affected since mortality was not an endpoint measurement.

### 2.8. Vaccination Formulation and Immunization

BP vaccines were either prepared without adjuvant or with Quil-A^®^ adjuvant (InvivoGen San Diego, CA, USA) at 20 μg/dose. The final *S. suis* antigens’ concentration in all vaccine formulations was 6 μg/dose. Empty BPs were used as negative control. Five-week-old female C57BL/6 mice (Charles River, Wilmington, MA, USA) were acclimatized to standard laboratory conditions with free access to water and rodent chow. Female mice were used in this study because they have been widely used in *S. suis* vaccine studies, which allows comparison with these studies [41]. Three animal trials were performed. In the first animal trial, mice (*n* = 10) were immunized subcutaneously (SC) with either two or three doses of 38-BP-Enol or SSU1-BP-SSU2 vaccines without adjuvant. Control (placebo) mice received three doses of control BPs. Sera was collected by cardiac puncture at Day 28 (two-dose groups) or at Day 35 (three-dose groups) for antibody titration by ELISA (see below). Challenge infection was not performed in the first trial. In the second trial, mice (*n* = 10) were immunized SC with two doses of 38-BP-Enol or SSU1-BP-SSU2 vaccines with Quil-A^®^ adjuvant. Control (placebo) mice received two doses of control BPs in Quil-A^®^. A group of naïve mice (PBS only) was also included. Sera was collected from the submandibular vein at Day 28 for antibody titration by ELISA. Challenge infection was performed at Day 28 by intraperitoneal (IP) injection of 8 × 10^7^ CFU/mL of *S. suis* serotype 2 strain P1/7. In the third trial, immunization was performed as described for the second trial, but challenge infection was performed with an infectious dose of 5 × 10^8^ CFU/mL. All immunizations were performed at 14-day intervals.

### 2.9. Mice Challenge Study

After challenge, infected mice were monitored for 10 days at least three times daily for mortality and clinical signs of systemic disease, such as rough hair coat, swollen/closed eyes, ocular oedema, hunchback, depression, lethargy, and clinical signs of meningitis such as spatial disorientation, hyperexcitation, opisthotonos, or circular walk with head towards one side. Severely affected animals were humanely euthanized.

### 2.10. Immunological Evaluation

Mouse sera were collected via cardiac puncture or submandibular bleeding for ELISA and immunoblot analyses. Sera were stored at −80 °C until analysis.

#### 2.10.1. Enzyme-Linked Immunosorbent Assays (ELISA)

Nunc-Immuno Polysorp low-binding plates (Thermo Scientific, Mississauga, ON, Canada) were coated overnight at 4 °C with 100 µL of 5 µg/mL of either the control BPs, 38-BP-Enol, or SSU1-BP-SSU2 diluted in phosphate-buffered saline (PBS buffer; pH 7.5). The next day, the plates were washed and blocked with 200 µL of 2% (*w*/*v*) bovine serum albumin (BSA) (HyClone, Logan, UT, USA) in PBS for 1 h at room temperature (RT) in a humidified chamber. After washing, 100 µL of mouse serum samples serially diluted in PBS 1% BSA were added to the wells and left for 1 h at RT in a humidified chamber. After washing, antibodies were detected using either HRP-conjugated goat anti-mouse Ig [IgG + IgM] diluted at 1:4000 or goat anti-IgG (Fcγ fragment specific; Jackson Immunoresearch, PA, USA) diluted at 1:4000 for 1 h at RT in a humidified chamber. Plates were developed with 3,3,5,5-tetramethylbenzidine (TMB; InvitroGen, Burlington, ON, Canada) substrate and the enzyme reaction was stopped by addition of 0.5 M H_2_SO_4_. Absorbance was read at 450 nm using a BioTek Synergy LX microplate reader (BioTek, Winooski, VT, USA). For mouse serum titration, the reciprocal of the last serum dilution that resulted in an OD_450_ of ≤ 0.15 (cutoff) was considered the titer of that serum.

#### 2.10.2. Immunoblot

To confirm the specificity of the IgG responses, immunoblot analysis was done. Pooled mouse sera from the vaccinated mice (*n* = 10) were diluted at 1/2000 and used against BP vaccines after SDS-PAGE run and transfer to nitrocellulose membranes (Life Technology, Waltham, MA, USA). The membrane was incubated with anti-mouse IgG HRP-conjugate (Abcam, Cambridge, UK) secondary antibody diluted at 1/20,000 for detection of bound IgG antibodies. For detection, the signal was developed using SuperSignal West Pico Stable Peroxide Solution and SuperSignal West Pico Luminol/Enhancer Solution (Thermo Scientific). The blots were imaged using the Odyssey^®^ Fc Imaging System (LI-COR^®^, Lincoln, NE, USA).

### 2.11. Statistical Analysis

Data are expressed as individual values, with horizontal bars representing the mean of the group. Data were analyzed by *t*-test with Mann–Whitney. The Kaplan–Meier method and log-rank Mantel–Cox tests were used to compare the survival rates of the mouse studied groups.

## 3. Results

### 3.1. Design of BPs Displaying S. suis Antigens as Particulate Vaccines

To develop particulate vaccine against *S. suis* diseases, we utilized an endotoxin-free *E. coli* ClearColi^TM^ BL21 (DE3) strain [39] to generate BPs coated with *S. suis* antigens at high copy number by employing the strong T7 promoter. Furthermore, the use of ClearColi^TM^ BL21 (DE3) strain eradicates laborious endotoxin removal processes, facilitating cost-effective manufacture of the vaccines for veterinary and human applications.

**Figure 1:**
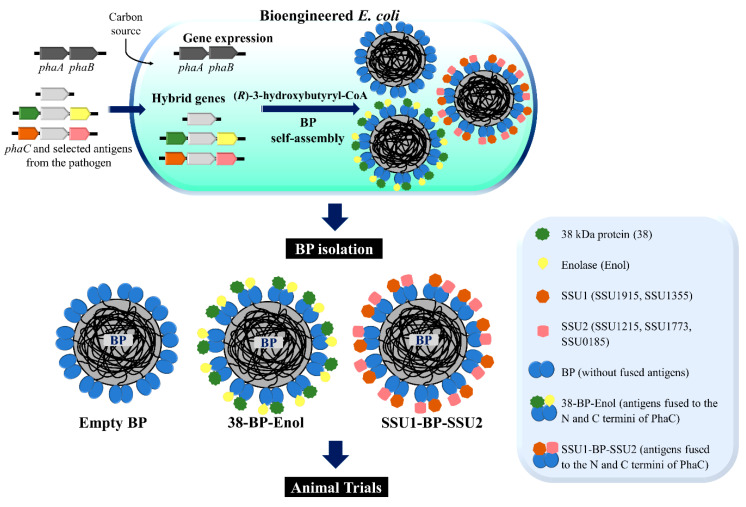
Development of biopolymer particles (BPs) displaying *S. suis* antigens as particulate vaccines. A schematic overview of the production of BPs displaying *S. suis* antigens produced from recombinant *E. coli* ClearColi^TM^ BL21 (DE3) production strain. The strains were genetically modified for assembly of antigen-coated BPs. BPs were isolated by mechanical disruption and subsequent downstream processing. PhaC is the PHB synthase. PhaA is acetyl-CoA acetyltransferase. PhaB is acetoacetyl-CoA reductase.

Figure 1 shows a schematic overview of the design and production of the BPs displaying the selected antigens, 38 kDa protein and enolase (38-BP-Enol), and the five recently proposed vaccine candidate antigens, SSU1915, SSU1355, SSU0185, SSU1215, and SSU1773 (SSU1-BP-SSU2), as particulate vaccines. Three enzymes were used for BP production, PhaC, PhaA, and PhaB. The seven candidate antigens that had shown vaccine potential in previous studies [11,14,15] were selected for bioinformatics analysis to predict antigenic epitopes (Table 1). Table 1 shows the antigens and predicted epitopes as well as their function and immune responses in mice in previous studies. The predicted epitopes of the seven proteins were translationally fused to PhaC to enable production of respective BPs (Figure 1, Table 1). We designed BPs with PhaC molecules each anchoring six copies of each of the predicted epitopes of 38 and enolase and three copies each of the predicted epitopes of SSU1915, SSU1355, SSU0185, SSU1215, and SSU1773 to the BPs. The differences in repeats were dictated by the different numbers of epitopes per BP as BP production shows increasingly lower yields with increasing epitope chain length.

### 3.2. In Vivo Self-Assembly of BP Vaccines and Their Characterization

**Figure 2:**
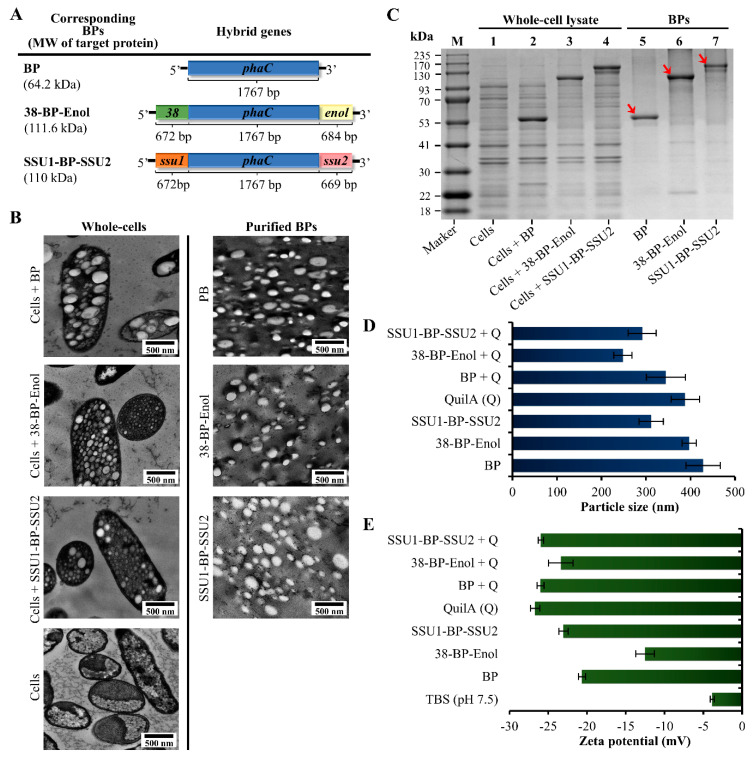
Physical and biochemical characterization of various biopolymer particles (BPs) displaying *S. suis* antigens. (**A**) Schematic representation of hybrid genes encoding fusion proteins for the production of empty BP, 38-BP-Enol, and SSU1-BP-SSU2 produced in recombinant *E. coli* ClearColi^TM^ BL21 (DE3) strain. MW (Molecular weight). The *phaC* is the PHB synthase gene. (**B**) Production and accumulation of various BPs were analyzed by TEM in whole-cell and purified BP fractions. (**C**) Protein profile analysis of whole-cell lysate and the purified BPs separated by SDS-PAGE and gel stained with Coomassie Blue. PhaC (64.2 kDa), 38-PhaC-Enol (111.6 kDa), and SSU1-PhaC-SSU2 (110 kDa) fusion proteins coating BP, 38-BP-Enol, and SSU1-BP-SSU2, respectively, are shown in red arrows. Protein identity was confirmed by Q-TOF-MS (Table S2). (**D**) Size of BPs before and after formulation with Quil-A^®^ adjuvant. (**E**) Zeta-potential of BPs before and after formulation with Quil-A^®^ adjuvant. The particle size and zeta-potential of each BP were measured three times by Zetasizer Nano ZS. Each data point of measurement represents the mean ± the standard error of the mean. The values are shown in Table S3.

*E. coli* was genetically modified by introducing hybrid genes encoding fusion proteins of the selected antigen(s)/epitopes fused to the BP anchoring domain, the PHB synthase (Figure 2A, Table S1). Recombinant *E. coli* harboring respective plasmids overexpressed the integrated hybrid genes *phaC* (empty BP production as negative control), *38-phaC-enol* (38-BP-Enol production), and *ssu1-phaC-ssu2* (SSU1-BP-SSU2 production). Overexpression of the genes enabled BP core synthesis and dense coating of BPs with respective antigens.

Physical and biochemical characterizations were done for the various BPs and the attached antigens/epitopes (Figure 2). TEM images showed accumulation of the BPs inside *E. coli* as well as the morphology of BPs before and after isolation and purification (Figure 2B). The images show the production of spherically shaped BPs inside the cell and preservation of their shape after purification [35,42]. Furthermore, the BPs showed a size distribution within the same sample, i.e., they were polydisperse, as reported earlier [23,35,40]. Synthesis of BP particles is based on the native, highly processive polymer synthase, resulting in presumably more polymer production when compared to the polymer synthase fused to various antigens. SDS-PAGE was done to analyze the protein profiles of the whole-cell lysates and purified BPs (Figure 2C). Full-length fusion proteins and dominating protein bands corresponded to the theoretical molecular weights of PhaC (64.2 kDa) and 38-PhaC-Enol (111.6 kDa) coating BP and 38-BP-Enol, respectively. However, SSU1-PhaC-SSU2 (110 kDa) coating SSU1-BP-SSU2 was higher than its theoretical molecular weight. Hence, analysis for detection of tryptic peptides was used to confirm the presence of SSU1-PhaC-SSU2 fusion protein coating the SSU1-BP-SSU2 beads (Table S2). Additionally, PhaC and 38-PhaC-Enol proteins coating BP and 38-BP-Enol were confirmed using Q-TOF-MS. These data confirmed successful bioengineering of *E. coli* for production of various BPs coated with selected *S. suis* antigens/epitopes. The fusion proteins were quantified by densitometry analysis as a percentage of the total protein in BP fractions (20% (*w*/*v*)) using BSA standards. Results showed that PhaC, 38-PhaC-Enol, and SSU1-PhaC-SSU2 accounted to 0.280, 0.174, and 0.172 µg/µL, respectively (Figure S2).

BP vaccines were formulated with Quil-A^®^ in preparation for animal vaccination studies. The impact of Quil-A^®^ on BPs was analyzed. The Zetasizer Nano ZS was used to determine the effect of Quil-A^®^ on size and zeta-potential of the BPs. The measurements were done before and after the addition of Quil-A^®^ adjuvant. The various BPs were polydisperse (>0.1) and particle sizes ranged between 312–428 nm before formulation with Quil-A^®^ (Figure 2D; Table S3). These measurements are consistent with the TEM analysis (Figure 2B). These results were aligned with size distributions were previously described for BPs [18,35,43,44]. The formulation of various BPs with Quil-A^®^ adjuvant resulted in similar particle sizes, 248–344 nm, presumably due to the size of Quil-A^®^ (388 nm) itself that is similar to BPs. The surface charges of various BPs were negative at neutral pH due to the theoretical isoelectric points (pI) of PhaC (6.04 pI), 38 (6.51 pI), enolase (5.03 pI), SSU1 (4.33 pI), and SSU2 (6.62 pI) proteins coating the respective BPs. The surface charge of the BPs remained negative upon addition of Quil-A^®^ due to the negative charge of Quil-A^®^ itself.

### 3.3. Immunological Properties of BP Vaccines

**Figure 3:**
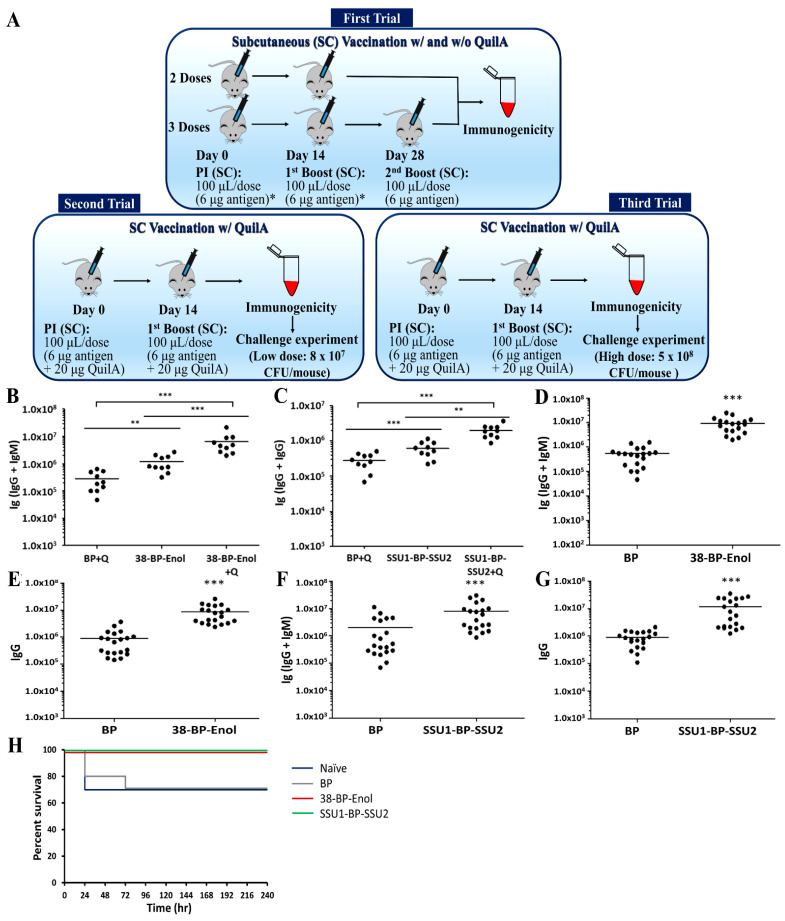
Immunological analysis and challenge of mice vaccinated with biopolymer particles (BPs) displaying *S. suis* antigens. (**A**) Schematic overview of the three animal trials. * With and without 20 μg/dose Quil-A^®^. (**B**,**C**) Results from the first animal trial. (**B**) Ig [IgG + IgM] response induced by 38-BP-Enol vaccination. (**C**) Ig [IgG + IgM] response induced by SSU-BP-SSU vaccination. Mice (n = 10) were immunized with two doses of 38-BP-Enol (6 µg/dose) and SSU1-BP-SSU2 (6 µg/dose) with adjuvant Quil-A^®^ (Q) or without adjuvant. BP + Q control mice received two doses of empty BPs + Quil-A^®^. Sera (day 28) from BP + Q or two-dose vaccination (38-BP-Enol or 38-BP-Enol + Q, SSU1-BP-SSU2, or SSU1-BP-SSU2 + Q) were analyzed by ELISA using either BP or 38-BP-Enol or SSU1-BP-SSU2 as coating antigen. Each dot represents a single vaccinated mouse, with horizontal bars representing the mean of the group; *p* values for each case tested were (**B**) *** *p* < 0.0001, *** *p* < 0.0001, ** *p* < 0.005 and (**C**) *** *p* < 0.0001), ** *p* < 0.007, *** *p* < 0.0001. (**D**,**G**) Combined ELISA results from the second and third animal trials. ELISA plates (**D**,**E**) were coated with antigen 38-BP-Enol, while F–G were coated with antigen SSU1-BP-SSU2. (**D**) Ig [IgG + IgM] response induced by 38-BP-Enol vaccination. (**E**) IgG response induced by 38-BP-Enol vaccination. (**F**) Ig [IgG + IgM] response induced by SSU1-BP-SSU2 vaccination. (**G**) IgG response induced by SSU1-BP-SSU2 vaccination. Mice were immunized with two doses of 38-BP-Enol (6 µg/dose) and SSU1-BP-SSU2 (6 µg/dose) with Quil-A^®^ adjuvant. (**D**,**G**) are combined results of second and third animal trials. Statistical analysis was performed using unpaired *t* tests Mann–Whitney from day 28 mouse sera; *p* values for each case tested were (**D**) *** *p* < 0.0004, (**E**) *** *p* < 0.0001, (**F**) *** *p* < 0.0001, and (**G**) *** *p* < 0.0001. Each dot represents a single vaccinated mouse (*n* = 20 mice per group), with horizontal bars representing the mean of the group. (**H**) Survival of vaccinated C57BL/6 mice with two doses of BP vaccines formulated with Quil-A^®^ after challenge with 8 × 10^7^ CFU/mouse of *S. suis* serotype 2 virulent strain P1/7. Representative of one experiment with 10 mice per group. Challenge result from the second animal trial.

In this study, three animal trials were conducted (Figure 3A). Ten female C57BL/6 mice per group were vaccinated two or three times SC at 14-day intervals with vaccines containing 6 µg of *S. suis* antigens/dose, formulated or not with Quil-A^®^ (20 µg per dose) in a total volume of 100 µL. The first animal trial tested the number of doses of various BP vaccines, i.e., two or three doses, to assess the relevance of the number of doses in the absence of adjuvant for induction of a significant immune response. The second animal trial involved the addition of Quil-A^®^ adjuvant and two doses of the BP vaccines to investigate immunogenicity and protective immunity as assessed by challenge with *S. suis*. The third animal trial repeated the second trial but using a challenge experiment with a more aggressive bacterial dosage (5 × 10^8^ CFU/mouse versus 8 × 10^7^ CFU/mouse).

**Figure 4:**
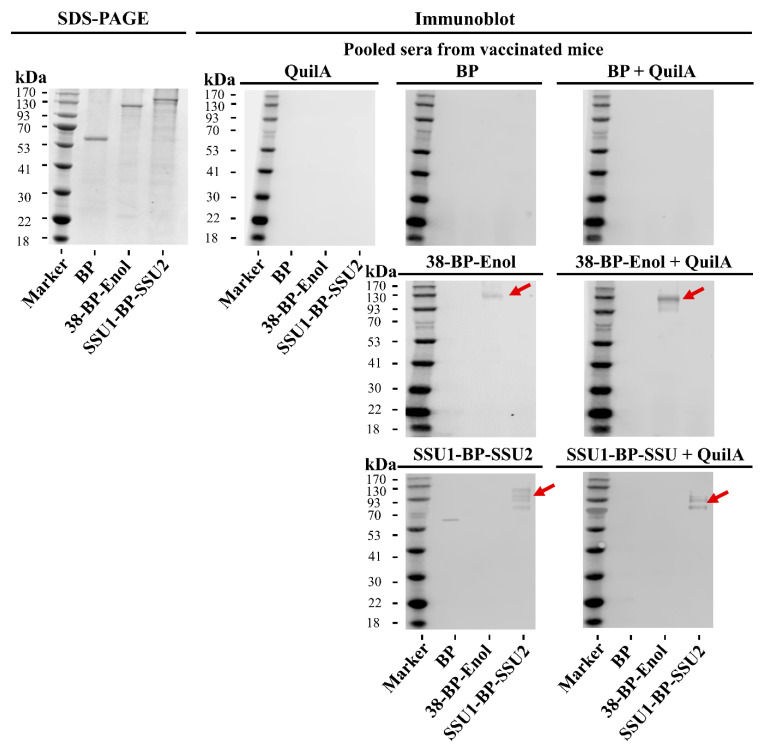
BP vaccines induce specific immune responses to the incorporated antigens. Specific antigen recognition was analyzed by immunoblotting using pooled sera from mice (*n* = 10 per group) vaccinated with BPs. The 38-BP-Enol and SSU1-BP-SSU2 are shown in red arrows.

In the first animal study, two and three doses of the BP vaccines were tested and resulted in similar immune responses; hence, two doses, which would be more desirable for a future vaccine product, were chosen to proceed to second and third animal trials for immunogenicity and challenge experiments (Figure S3). Furthermore, the results from the first animal trial (Figure 3A–C) indicated that BP vaccines were self-adjuvanting and induced significant specific immune responses in the absence of adjuvant. However, addition of Quil-A^®^ adjuvant significantly increased immune responses; hence, Quil-A^®^ adjuvant was added to vaccine formulations to increase the potential for induction of protective immunity. For immunogenicity results, the data were similar for the second and third animal studies (Figures S4 and S5); hence, results were combined in Figure 3D–G to enhance statistical power by increasing the group size to 20 mice. Ig [IgG + IgM] and IgG were measured by ELISA to characterize antigen-directed humoral immune responses. The 38-BP-Enol and SSU1-BP-SSU2-specific Ig and IgG immune responses were detected in mice vaccinated with 38-BP-Enol (Figure 3D,E) and SSU1-BP-SSU2 (Figure 3F,G). There was a significant induction of Ig and IgG in 38-BP-Enol and SSU1-BP-SSU2 vaccinated mice compared to the negative control empty BP group. Naïve serum resulted in titers (1.0 × 10^3^) significantly lower compared to the serum from empty BPs and BPs coated with antigens-vaccinated mice. These results suggested induction of humoral immune responses in mice vaccinated with 38-BP-Enol or SSU1-BP-SSU2. In addition, the specificity of the IgG responses was analyzed by immunoblotting using pooled sera from vaccinated mice. Reactivity of antibodies was tested against antigens present in 38-BP-Enol and SSU1-BP-SSU2 formulations and negative controls, Quil-A^®^, empty BP, and empty BP+Quil-A^®^ (Figure 4). While the specific antigens were detected by the pooled sera from mice vaccinated with corresponding 38-BP-Enol, 38-BP-Enol + Quil-A^®^, SSU1-BP-SSU2, and SSU1-BP-SSU2 + Quil-A^®^, antigens did not react with any antibodies induced in mice vaccinated with the negative controls Quil-A^®^, empty BP, and empty BP + Quil-A^®^ groups.

The protective efficacy of the 38-BP-Enol and SSU1-BP-SSU2 bead vaccines, respectively, was determined using a murine model. *S. suis* serotype 2 virulent strain P1/7 was used to challenge vaccinated animals because this is the most prevalent serotype to infect both pigs and humans [5,45]. Figure 3H shows 100% survival of the both 38-BP-Enol and SSU1-BP-SSU2 vaccinated mice, while there was 70% survival of the empty BP, Quil-A^®^, and buffer only (Naïve) vaccinated mice. The BP vaccines did not induce detectable protective immunity in the more aggressive bacterial challenge experiment using 6.25-fold increase number of the pathogen (Figure S4). However, the more realistic scenario of infection, i.e., exposure to a lower number of *S. suis* cells, is reflected in the second animal trial, where there was 100% survival of mice vaccinated with 38-BP-Enol and SSU1-BP-SSU2.

## 4. Discussion

In this study, we selected seven conserved antigens that had shown good protective immunity [11,14,15] (Figure 1; Table 1). We developed *S. suis* particulate vaccine candidates applying the BP vaccine technology that harnesses the capability of the bacteria to produce protein-coated BP inclusions [16,19]. Translational fusions of the various antigens to the BP-anchoring protein, PhaC, mediated production of antigen-coated BPs in endotoxin-free *E. coli*. Stability, cost-effective manufacture, endotoxin-free production, and high-density display of antigens make the BP technology attractive for vaccine development [18,46].

Characterization of BP beads confirmed the size range of BPs (312–428 nm without QuilA adjuvant; 292–344 nm with QuilA adjuvant) that is considered to be suitable for efficient uptake by antigen-presenting cells (APCs) (Figure 2B,D; Table S3). APCs take up particles with sizes of <5–10 μm via phagocytosis [43,44], facilitating antigen cross-presentation and induction of both humoral and cell-mediated immunity [16,47]. The zeta-potential of BPs was negative before and after formulation with the Quil-A^®^ adjuvant (Figure 2E, Table S3). Particles with positive zeta-potential demonstrated good protein absorption while negatively charged particles did not show substantial protein absorption [48]. In addition, the zeta-potential is an indicator of stability of particles (i.e., a higher charge on the surface of particle would prevent aggregation). In the case of combined electrostatic stabilization such as the BPs and Quil-A^®^ adjuvant, ±20 mV is desired and repulsion between particles with comparable electric charge avoids aggregation of the particles and, thus, stabilizes dispersions [48]. In this study, although Quil-A^®^ adjuvant absorption to BPs was not tested, results in Figure 3B,C showed that addition of Quil-A^®^ to BP vaccines significantly enhanced induction of specific antibodies. The zeta-potential values of Quil-A^®^-formulated BP vaccines were stable and close to the desirable value of ±20 mV. Further characterization showed that BPs were densely coated with full-length fusion proteins comprising the target antigens/epitopes (Figure 2C; Table S2).

Our vaccination studies in mice via SC administration confirmed that the 38-BP-Enol and SSU1-BP-SSU2 vaccines were immunogenic, i.e., induced significant and antigen-specific antibody responses (Figure 3 and Figure 4; Figures S3, S5 and S6). Two doses of the BP vaccines and addition of Quil-A^®^ adjuvant were found sufficient for induction of immune responses (Figure 3; Figures S2, S3, S5 and S6). Potential omission of adjuvants due to self-adjuvanting properties of BPs makes the vaccine formulation safer and more cost effective (Figure 3B,C). The 38-BP-Enol and SSU1-BP-SSU2 vaccines showed reproducible vaccine performance across three animal trials. Results of the immunogenicity studies from three animal trials showed that 38-BP-Enol and SSU1-BP-SSU2 vaccines induced humoral immune responses that might contribute to protective immunity against the most prevalent *S. suis* serotype 2 infection (Figure 3H) [5,45]. Optimization of the formulation and further studies in pigs would allow a better characterization of this vaccine platform.

*S. suis* is increasingly problematic due to the rise of antimicrobial resistance. *S. suis* is considered a niche for antibiotic resistance. Moreover, it represents a high risk of transmission of such resistance to other veterinary and human pathogens due to the presence of mobile genetic elements that carry resistance genes and that are transferable at high frequency within species and, even more alarming, between bacterial species [49]. Controlling *S. suis* serotype 2 infection in swine using methods other than antibiotics will thus be beneficial from both an economic point of view (swine production) and a “One-Health” perspective (human disease and antibiotic resistance gene spreading). In spite of numerous research studies on vaccine development against *S. suis*, no efficacious commercial vaccine is available [1]. Currently, only autogenous vaccines are used in the field and the results are conflicting [8]. Generally, autogenous vaccines are not tested for immunogenicity and protective efficacy, leading to considerable uncertainties concerning control of disease. Studies on identifying protective antigens of *S. suis* that can be used as subunit vaccines have shown some promise overcoming issues associated with autogenous vaccines [1,2]. However, there are still many issues to address, including the number of doses to be used, the cost effectiveness, the efficacy, and side effects. Several studies have shown induction of IgG antibody responses and some highlighted IgG1, IgG2, IgG2a, IgG2b, and IgG3 [1]. However, there is no clear understanding of what immune response is required for an effective *S. suis* vaccine. Overall, this study suggests that the BP vaccine technology can be adapted for design and manufacture of particulate subunit vaccines that might protect against *S. suis* infection.

## 5. Conclusions

In conclusion, BPs displaying the selected *S. suis* antigens induced protective immunity against acute mild infection by virulent *S. suis* serotype 2 strain P1/7. Antibody-mediated immunity likely played an important role toward protective immunity. In this study, two BP vaccines incorporating either the 38 kDa protein and the enolase or the novel five antigens SSU1915, SSU1355, SSU0185, SSU1215, and SSU1773 were produced as particulate vaccines. BP vaccines were produced at high yield, suggesting cost-effective and scalable manufacturability. Future studies should include evaluation of the BP vaccine candidates in the pig model of *S. suis* infection.

## Figures and Tables

**Table 1:** Antigens and predicted epitopes for design of 38-BP-Enol and SSU1-BP-SSU2 particulate vaccines.

Antigens	Predicted Epitopes *	Function	Immune Response in Mice	Reference
38 kDa protein	RIIKETAEASDMGA (×6); KRALEKVRGESFDV (×6)	Unknown	Antibody response	[11]
Enolase	CASSEFYDKERKVY (×6); KFEGEGAAVRTSAE (×6)	Glycolytic enzyme, binds to plasminogen of an infected host	IgG antibodies	[14]
SSU1 (SSU1915, SSU1355) SSU2 (SSU0185, SSU1215, SSU1773)	SSU1915 (Putative maltose/maltodextrin-binding protein precursor (Ma1X): QLSELTLADDSKAD (×3), DATNEVPANTEARE (×3); SSU1355 (Putative surface-anchored 5′-nucleotidase): AETETPAESIRVQA (×3), APISNKKTEKASGN (×3); SSU0185 (Putative tagatose-6-phosphate aldose/ketose isomerase (AgaS): CNNLPDTPSPTGTV (×3); SSU1215 (Putative surface-anchored dipeptidase): PSDKKVTPTNKKGK (×3); SSU1773 (Putative surface-anchored serine protease): TTAGKTTDESKEKE (×3), KDLRINTSPESLDE (×3)	SSU1915: carbohydrate metabolism, specifically, in polysaccharide degradation and synthesis; SSU1355: hydrolytic enzyme; SSU0185: part of pathway for utilization of the amino sugar *N*-acetyl-d-galactosamine; SSU1215: catabolism of exogenously supplied peptides and the final steps of protein turnover; SSU1773: cell signalling	Ig [IgG + IgM] antibodies	[15]

* Antigenic epitopes were predicted by antigenicity analysis (Jameson–Wood guide), surface probability (Emni method) and hydrophilicity (Kyte–Doolittle). Furthermore, the predicted epitopes were confirmed by Bepipred, BCPred, AAP, and FBCPred.

## Data Availability

The data are available under reasonable request to the corresponding authors.

## Supplementary Materials

The following are available online at https://www.mdpi.com/article/10.3390/vaccines9121386/s1. Table S1: Bacterial strains, plasmids, and primers used in this study. Figure S1: Plasmid construction of pET14b_38-PhaC-Enol and pET14b_SSU1-PhaC-SSU2 for 38-BP-Enol and SSU1-BP-SSU2 production in E. coli strain ClearColiTM, respectively. Table S2: Q-TOF-MS analysis of PhaC and PhaC-fusion proteins. Figure S2: Protein quantification of the purified BP vaccines displaying *S. suis* antigens by densitometry. Table S3: Particle size and zeta-potential measurements of the BP bead vaccines. Figure S3: Two doses versus three doses of BP vaccines. Figure S4: Ig (IgG + IgM) response induced by BP vaccines for the second and third animal trials. Figure S5: IgG response induced by BP vaccines for the second and third animal trials. Figure S6: Survival of immunized C57BL/6 mice with two doses of vaccines + QuilA after challenge with 5 × 10^8^ CFU/mouse of *S. suis* serotype 2 virulent strain P1/7.
