# Supplementary material for: Engineering Antigens to Assemble into Polymer Particle Vaccines for Prevention of Streptococcus suis Infection
Source: Vaccines (Basel). 2021 Nov 24;9(12):1386. doi: 10.3390/vaccines9121386 (PMC8709461; doi:10.3390/vaccines9121386)
Supplement: Supplementary file 1 [file vaccines-09-01386-s001.zip › vaccines-1431910-supplementary.pdf]

vaccines

Supplementary Material

Engineering antigens to assemble into polymer particle vaccines for prevention of Streptococcus suis infection

Zennia Jean C. Gonzaga [1], Shuxiong Chen [1], Mélanie Lehoux [2], Mariela Segura [2] and Bernd H. A. Rehm [1,3,*]

1 Centre for Cell Factories and Biopolymers (CCFB), Griffith Institute for Drug Discovery, Griffith University, Don Young Road, Nathan QLD 4111, Australia; jean.gonzaga@griffithuni.edu.au (Z.J.C.G.); shuxiong.chen@griffith.edu.au (S.C.)

2 Research Group on Infectious Diseases in Production Animals and Swine and Poultry Infectious Diseases Research Centre, Faculty of Veterinary Medicine, Université de Montréal, 3200 rue Sicotte, CP5000, St-Hyacinthe, QC J2S 7C6, Canada; melanie.lehoux@umontreal.ca (M.L.); mariela.segura@umontreal.ca (M.S.)

3 Menzies Health Institute Queensland (MHIQ), Griffith University, Gold Coast, Qld 4222, Australia

* Correspondence: b.rehm@griffith.edu.au; Tel.:+61737354233

Table S1. [illegible] strains, plasmids and primers used in this study.

**Strains, Plasmids and Primers**	**Relevant characteristics**	**References**
**1. Bacterial strains**		
E. coli		
XL1-Blue	*recA1 endA1 gyrA96 thi-1 hsdR17 supE44 relA1 lac* [F′ *proAB lacI*q *lacZ* Δ*M15* Tn*10* (Tetr)]	Stratagene
ClearColiTM BL21(DE3)	F– *ompT hsdSB (rB- mB-) gal dcm lon* λ(DE3 [*lacI lac*UV5-T7 gene 1 *ind1 sam7 nin5*]) *msbA148* Δ*gut*QΔ*kds*D Δ*lpxL*Δ*lpxM*Δ*pagP*Δ*lpxP*Δ*eptA*	Lucigen
S. suis		
S. suis serotype 2 strain P1/7	Challenge strain in this study that was isolated from a field case of meningitis in a pig	[1]
**2. Plasmids**		
pET14b	Amp^R; T7 promoter	Novagen
pMCS69	Cm^R; T7 promoter; pBBR1MCS derivative containing codon optimised genes *phaA* and *phaB* from *C. necator*	[2]
pET14b_PhaC-RV1626	pET-14b_PhaC derivative containing RV1626	[3]
pET14b_PhaC	pET-14b derivative containing *phaC* gene fragment	[4]
pUC57_38	pUC57 derivative containing *E. coli* codon optimized 38 fragment flanked by *Xba*I/*Spe*I sites	This study
pUC57_enol	pUC57 derivative containing *E. coli* codon optimized enol fragment flanked by *Xho*I/*BamH*I sites	This study
pET14b_PhaC-enol	Codon optimized enol fragment from pUC57_enol replacing RV1626 into *Xho*I/*BamH*I sites of pET14b_PhaC-RV1626	This study
pET14b_38-PhaC-enol	Codon optimized 38 fragment from pUC57_38 inserted into *Xba*I/*Spe*I sites of pET14b_PhaC-enol	This study
pUC57_SSU1	pUC57 derivative containing *E. coli* codon optimized SSU1 fragment flanked by *Xba*I/*Spe*I sites	This study
pUC57_SSU2	pUC57 derivative containing *E. coli* codon optimized SSU2 fragment flanked by *Xho*I/*BamH*I sites	This study
pET14b_PhaC-SSU2	Codon optimized enol fragment from pUC57_SSU2 replacing RV1626 into *Xho*I/*BamH*I sites of pET14b_PhaC-RV1626	This study
pET14b_SSU1-PhaC-SSU2	Codon optimized 38 fragment from pUC57_SSU1 inserted into *Xba*I/*Spe*I sites of pET14b_PhaC-SSU2	This study
**3. Primers**	5′-3′	
T7 promoter	TAATACGACTCACTATAGGG	GenScript, USA
T7 termiantor	GCTAGTTATTGCTCAGCGG	GenScript, USA

Amp^r, ampicillin resistance;; Tet^r, tetracycline resistance; Cm^r chloramphenicol resistance.

A

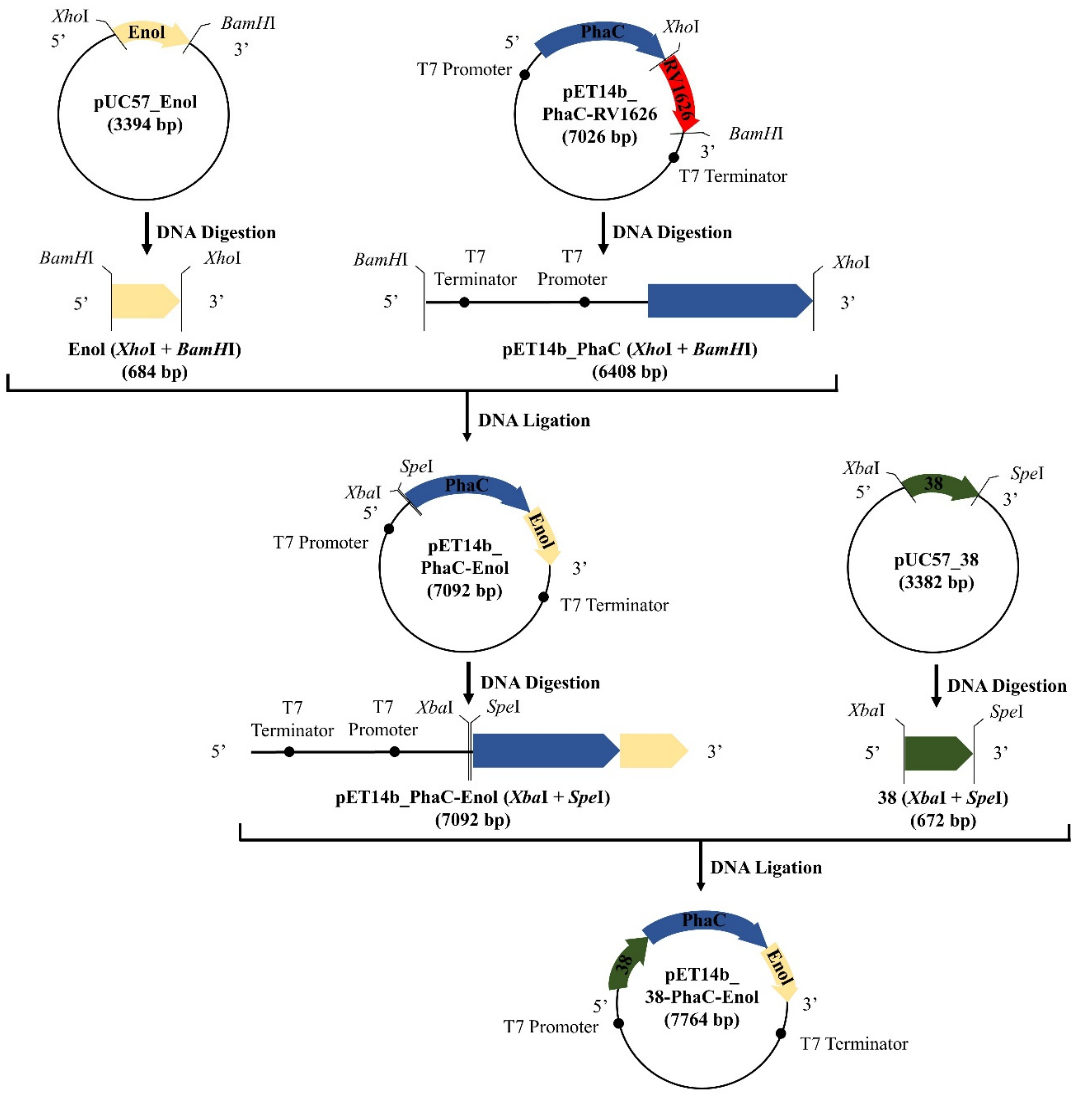
XhoI
Enol
BamHI
5'
3'
pUC57_Enol
(3394 bp)
PhaC
XhoI
T7 Promoter
pET14b_
PhaC-RV1626
(7026 bp)
RV1626
BamHI
T7 Terminator
DNA Digestion
Enol (XhoI + BamHI)
(684 bp)
T7
Terminator
T7
Promoter
pET14b_PhaC (XhoI + BamHI)
(6408 bp)
DNA Ligation
SpeI
XbaI
pET14b_
PhaC-Enol
(7092 bp)
38
pUC57_38
(3382 bp)
pET14b_PhaC-Enol (XbaI + SpeI)
(7092 bp)
38 (XbaI + SpeI)
(672 bp)
pET14b_
38-PhaC-Enol
(7764 bp)

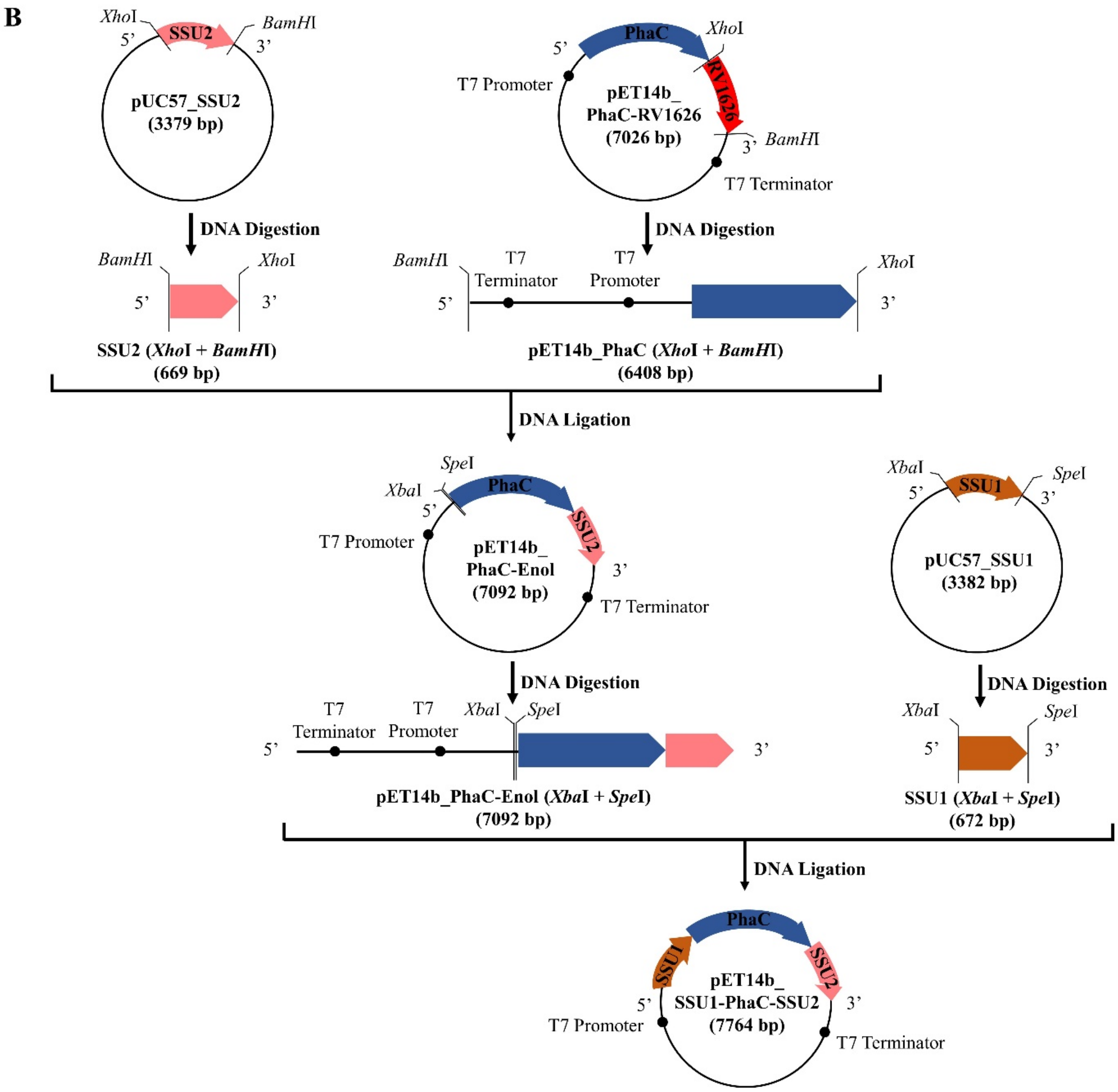

Figure S1. Plasmid construction of pET14b_38-PhaC-Enol and pET14b_SSU1-PhaC-SSU2 for 38-BP-Enol and SSU1-BP-SSU2 production in *E. coli* strain ClearColi™, respectively. (**A**) The DNA fragments encoding the *38* and *enol* genes were isolated from pUC57_38 and pUC57_Enol by DNA hydrolysis, respectively. The Enol fragment was introduced first to the 3′ end of PhaC by replacing RV1626 into *Xho*I/*BamH*I sites, resulting to pET14b_PhaC-Enol. Subsequently, the 38 fragment was inserted into the *Xba*I/*Spe*I sites to the 5′ end of PhaC-Enol, generating the final plasmid pET14b_38-PhaC-Enol. (**B**) The DNA fragments encoding the *SSU1* and *SSU2* genes were isolated from pUC57_SSU1 and pUC57_SSU2 by DNA hydrolysis, respectively. The SSU2 fragment was introduced first to the 3′ end of PhaC by replacing RV1626 into *Xho*I/*BamH*I sites, resulting to pET14b_PhaC-SSU2. Subsequently, the SSU1 fragment was inserted into the *Xba*I/*Spe*I sites to the 5′ end of PhaC-SSU2, generating the final plasmid pET14b_SSU1-PhaC-SSU2. PhaC is the BP synthase.

Table S2. Q-TOF-MS analysis of PhaC and PhaC-fusion proteins.

Protein Sequence*	Protein sequence coverage and the confirmed fragments
PhaC (MW: 64.2 kDa)<br>1 MATGKGAAASTQEGKSQPFKVTPGPFDPATWLEWSRQWQGTEGNGHAAAS<br>51 GIPGLDALAGVKIAPAQLGDIQQRYMKDFSALWQAMAEGKAEATGPLHDR<br>101 RFAGDAWRTNLPYRFAAAFYLLNARALTELADAVEADAKTRQRIRFAISQ<br>151 WVDAMSPANFLATNPEAQRLLIESGGESLRAGVRNMMEDLTRGKISQTDE<br>201 SAFEVGRNVAVTEGAVVFENEYFQLLQYKPLTDKVHARPLLMVPPCINKY<br>251 YILDLQPESSLVRHVVEQGHTVFLVSWRNPDASMAGSTWDDYIEHAAIRA<br>301 IEVARDISGQDKINVLGFCVGGTIVSTALAVLAARGEHPAASVTLLTTLL<br>351 DFADTGILDVFVDEGHVQLREATLGGGAGAPCALLRGLELANTFSFLRPN<br>401 DLVWNYVVDNYLKGNTPVPFDLLFWNGDATNLPGPWYCWYLRHTYLQNEL<br>451 KVPGKLTVCGVPVDLASIDVPTYIYGSREDHIVPWTAAYASTALLANKLR<br>501 FVLGASGHIAGVINPPAKNKRSHWTNDALPESPQQWLAGAIEHHGSWWPD<br>551 WTAWLAGQAGAKRAAPANYGNARYRAIEPAPGRYVKAKA	79%<br>V21-R74, D78-K90, F115-T140, F146-R180, N185-K234, Y250-R299, D306-R442, L456-K498, F501-K518, R521-562
38-PhaC-Enol (MW: 111.6 kDa)<br>1 MRIIKETAEASDMGA-LINKER-RIIKETAEASDMGA-LINKER-RIIKETAEASD<br>51 MGA-LINKER-RIIKETAEASDMGA-LINKER-RIIKETAEASDMGA-LINKER-RIIK<br>101 ETAEASDMGA-LINKER-KRALEKVRGESFDV-LINKER-KRALEKVRGESFDVGP<br>151 GPGKRALEKVRGESFDV-LINKER-KRALEKVRGESFDV-LINKER-KRALEKVRG<br>201 ESFDV-LINKER-KRALEKVRGESFDVSATGKGAAASTQEGKSQPFKVTPGPF<br>251 DPATWLEWSRQWQGTEGNGHAAASGIPGLDALAGVKIAPAQLGDIQQRYM<br>301 KDFSALWQAMAEGKAEATGPLHDRRFAGDAWRTNLPYRFAAAFYLLNARA<br>351 LTELADAVEADAKTRQRIRFAISQWVDAMSPANFLATNPEAQRLLIESGG<br>401 ESLRAGVRNMMEDLTRGKISQTDESAFEVGRNVAVTEGAVVFENEYFQLL<br>451 QYKPLTDKVHARPLLMVPPCINKYYILDLQPESSLVRHVVEQGHTVFLVS<br>501 WRNPDASMAGSTWDDYIEHAAIRAIEVARDISGQDKINVLGFCVGGTIVS<br>551 TALAVLAARGEHPAASVTLLTTLLDFADTGILDVFVDEGHVQLREATLGG<br>601 GAGAPCALLRGLELANTFSFLRPNDLVWNYVVDNYLKGNTPVPFDLLFWN<br>651 GDATNLPGPWYCWYLRHTYLQNELKVPGKLTVCGVPVDLASIDVPTYIYG<br>701 SREDHIVPWTAAYASTALLANKLRFVLGASGHIAGVINPPAKNKRSHWTN<br>751 DALPESPQQWLAGAIEHHGSWWPDWTAWLAGQAGAKRAAPANYGNARYRA<br>801 IEPAPGRYVKAKAHMVLAVAIDKRGGGGGLECASSEFYDKERKVY-LINKER-<br>851 CASSEFYDKERKVY-LINKER-CASSEFYDKERKVY-LINKER-CASSEFYDKERK<br>901 VY-LINKER-CASSEFYDKERKVY-LINKER-CASSEFYDKERKVY-LINKER-KFEGE<br>951 GAAVRTSAE-LINKER-KFEGEGAAVRTSAE-LINKER-KFEGEGAAVRTSAEGPG<br>1001 PGKFEGEGAAVRTSAE-LINKER-KFEGEGAAVRTSAE-LINKER-KFEGEGAAVR<br>1051 TSAE	41%<br>V122-R136, V141-R155, V160-R174, V179-R193, V198-R212, I287-R298, F326-R332, F339-R349, L394-G400, E401-R404, I419-R431, Y474-S500, W501-R523, E595-G600, G601-R610, A788-R797, R824-G850, C851-K900, V901-E950, G951-G1000, P1001-R1050
SSU1-PhaC-SSU2 (MW: 110 kDa)<br>1 MQLSELTLADDSKAD-LINKER-QLSELTLADDSKAD-LINKER-QLSELTLADDS<br>51 KAD-LINKER-DATNEVPANTEARE-LINKER-DATNEVPANTEARE-LINKER-DATN<br>101 EVPANTEARE-LINKER-AETETPAESIRVQA-LINKER-AETETPAESIRVQAGP<br>151 GPGAETETPAESIRVQA-LINKER-APISNKKTEKASGN-LINKER-APISNKKTE<br>201 KASGN-LINKER-APISNKKTEKASGNSATGKGAAASTQEGKSQPFKVTPGPF<br>251 DPATWLEWSRQWQGTEGNGHAAASGIPGLDALAGVKIAPAQLGDIQQRYM<br>301 KDFSALWQAMAEGKAEATGPLHDRRFAGDAWRTNLPYRFAAAFYLLNARA<br>351 LTELADAVEADAKTRQRIRFAISQWVDAMSPANFLATNPEAQRLLIESGG<br>401 ESLRAGVRNMMEDLTRGKISQTDESAFEVGRNVAVTEGAVVFENEYFQLL<br>451 QYKPLTDKVHARPLLMVPPCINKYYILDLQPESSLVRHVVEQGHTVFLVS<br>501 WRNPDASMAGSTWDDYIEHAAIRAIEVARDISGQDKINVLGFCVGGTIVS<br>551 TALAVLAARGEHPAASVTLLTTLLDFADTGILDVFVDEGHVQLREATLGG<br>601 GAGAPCALLRGLELANTFSFLRPNDLVWNYVVDNYLKGNTPVPFDLLFWN<br>651 GDATNLPGPWYCWYLRHTYLQNELKVPGKLTVCGVPVDLASIDVPTYIYG<br>701 SREDHIVPWTAAYASTALLANKLRFVLGASGHIAGVINPPAKNKRSHWTN<br>751 DALPESPQQWLAGAIEHHGSWWPDWTAWLAGQAGAKRAAPANYGNARYRA	76%<br>Q2-W255, Q263-F303, Q308-F326, R332-Y337, L345-R349, L351-R365, V377-R404, T415-F442, Q448-F497, R502-H519, R523-L540, C543-L571, D576-C606, R610-F618, L621-W628, L636-F644, N650-W661, Y665-Y698, G700-K744, T749-H756, L780-Y808, A818-R824, N834-T898, A948-E1051

801 IEPAPGRYVKAKAHMVLAVAIDKRGGGGGLECNNLPDTPSPTGTV-LINKER-
851 CNNLPDTPSPTGTV-LINKER-CNNLPDTPSPTGTV-LINKER-PSDKKVTPTNKK
901 GK-LINKER-PSDKKVTPTNKKGK-LINKER-PSDKKVTPTNKKGK-LINKER-TTAGK
951 TTDESKEKE-LINKER-TTAGKTTDESKEKE-LINKER-TTAGKTTDESKEKEGPG
1001 PGKDLRINTSPESLDE-LINKER-KDLRINTSPESLDE-LINKER-KDLRINTSPE
1051 SLDE

*Confirmed sequences are in red colour. MW (Molecular weight).

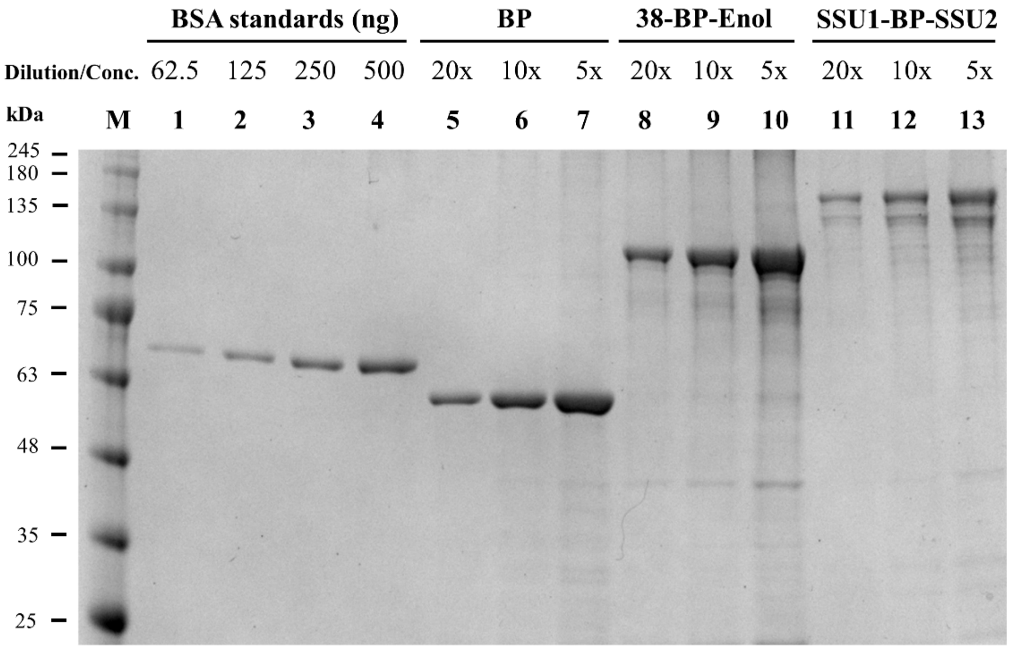

Band %	94.2	77.5	80.2
Antigen/protein (μg/μL)	0.280	0.174	0.172

Figure S2. Protein quantification of the purified BP vaccines displaying *S. suis* antigens by densitometry. Different amounts of BSA standard ranging between 62.5 and 500 ng were loaded on Bis-Tris gel to generate a standard curve, and were used to determine the antigen concentrations. The image was taken by the gel doc (BioRad Laboraties, Hercules, CA), and analysed with the Image Lab software (BioRad Laboratories, Hercules, CA). M, marker.

Table S3. Particle size and zeta-potential measurements of the BP bead vaccines.

BP beads	Ave. Size (nm)	Std. dev. (Size)	Ave. PDI	Ave. Zeta Potential (mV)	Std. dev. (Zeta Potential)
TBS buffer (pH 7.5)	-	-	-	-3.89	0.21
BP	428	38.12	0.36	-20.67	0.40
38-BP-Enol	397	15.36	0.35	-12.53	1.15
SSU1-BP-SSU2	312	27.54	0.30	-23.03	0.60
QuilA	388	31.37	0.59	-26.70	0.53
BP + QuilA	344	43.60	0.55	-26.00	0.40
38-BP-Enol + QuilA	248	19.98	0.44	-23.37	1.50
SSU1-BP-SSU2 + QuilA	292	30.88	0.51	-25.97	0.23

Ave. (Average); Std. dev. (Standard deviation); PDI (Polydispersity index).

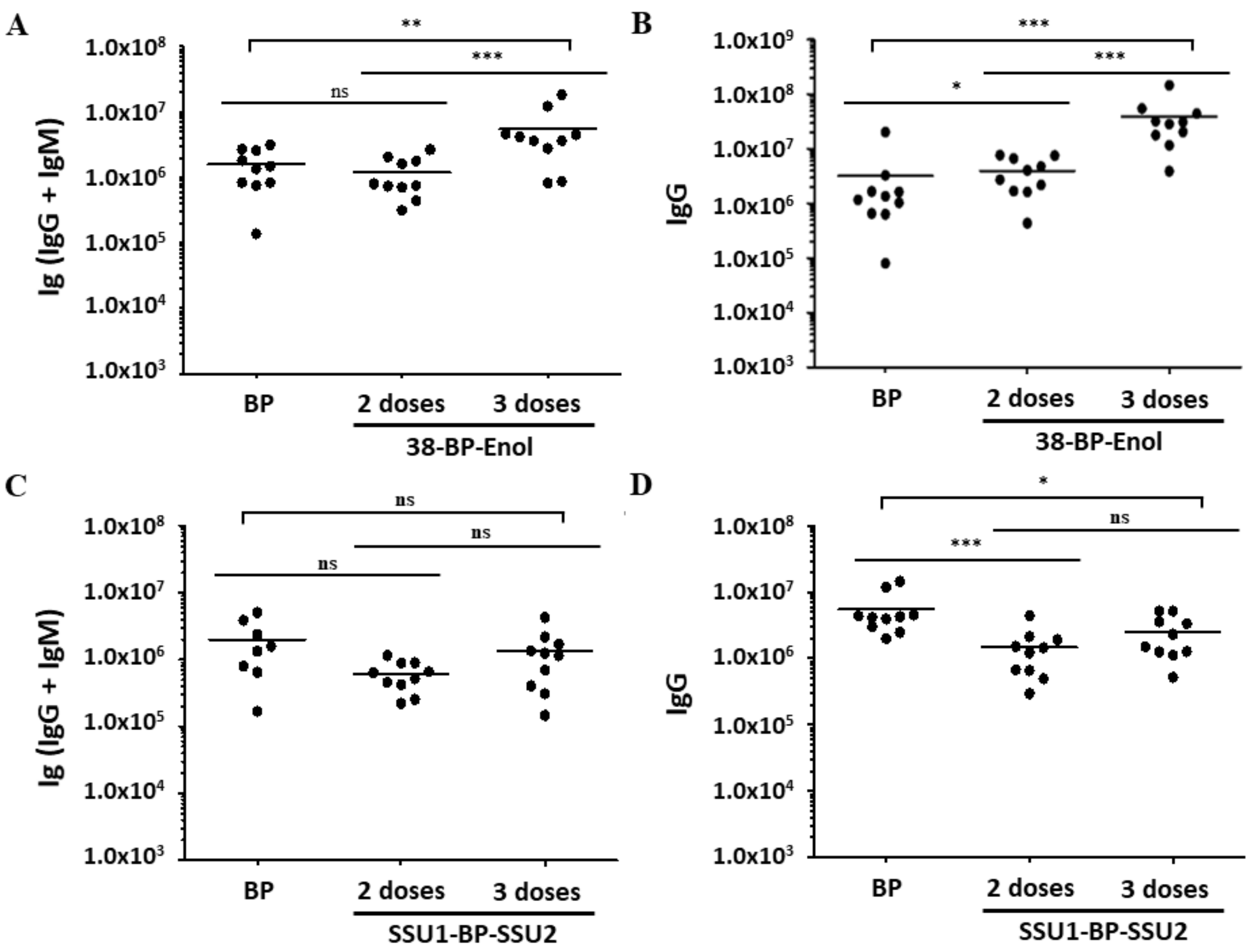

Figure S3. Two doses versus three doses of BP vaccines. (**A**) Ig [IgG + IgM] response induced by 38-BP-enol vaccination. (**B**) IgG response induced by 38-BP-Enol vaccination. (**C**) Ig [IgG + IgM] response induced by SSU-BP-SSU vaccination. (**D**) IgG response induced by SSU-BP-SSU vaccination. Mice (n = 10) were immunized with 2 or 3 doses of 38-BP-Enol (6 µg dose) and SSU1-BP-SSU2 (6 µg dose) without adjuvant. BP control mice received 3 doses. Sera (day 28) from BP, or 2-dose or 3-dose vaccination were analyzed by ELISA using either BP or 38-BP-Enol or SSU1-BP-SSU2 as coating. Statistical analysis was performed using unpaired t tests Mann–Whitney. Each dot represents a single vaccinated mouse (n = 10 mice per group), with horizontal bars representing the mean of the group. P values for each case tested were (**A**) **$p < 0.007$, ***$p < 0.0007$; (**B**) ***$p < 0.0001$,) ***$p < 0.0002$, *$p < 0.04$; and (**D**) *$p < 0.04$, ***$p < 0.0007$. ns means not statistically significant.

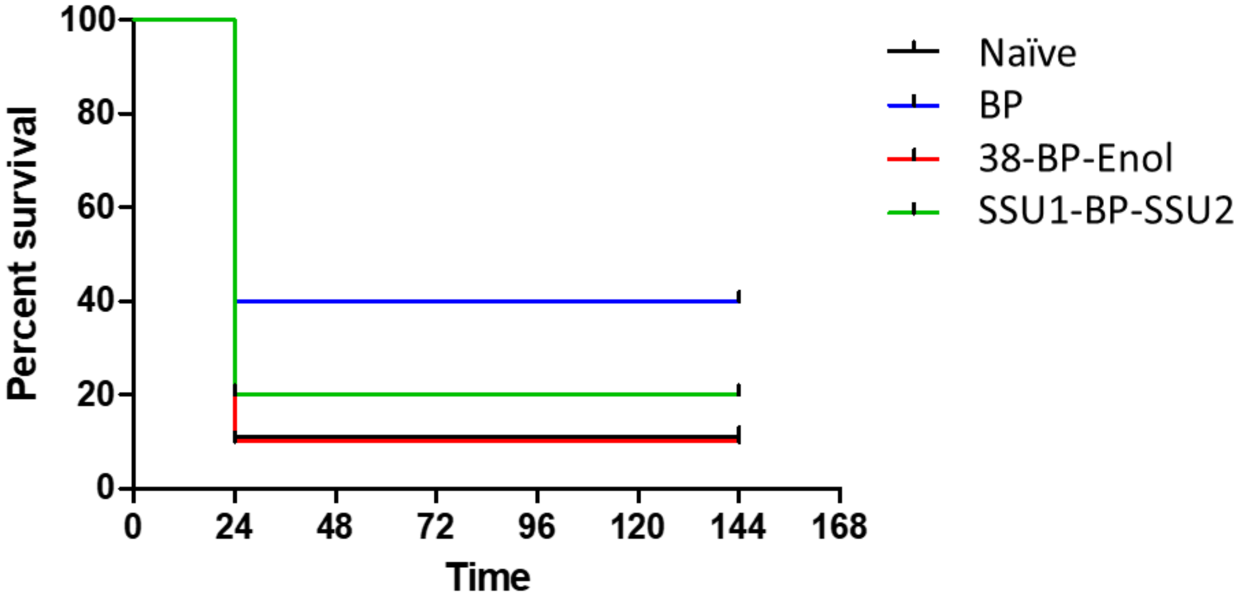

Figure S4. Survival of immunized C57BL/6 mice with two doses of vaccines + Quil-A® after challenge with 5 x 10^8 CFU/mouse of *S. suis* serotype 2 virulent strain P1/7. Representative of one experiment with 10 mice per group.

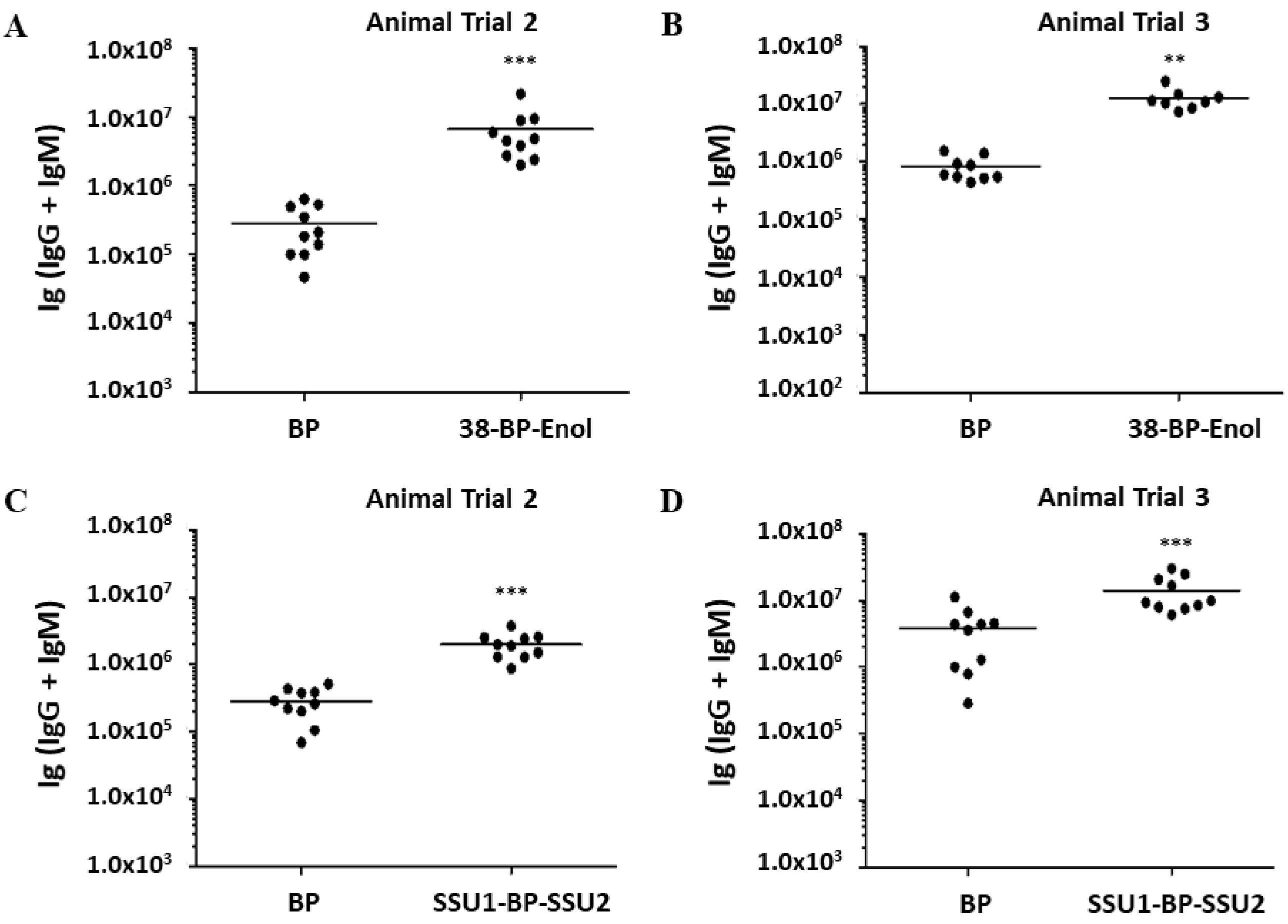

Figure S5. Ig [IgG + IgM] response induced by BP vaccines for the second and third animal trials. (A-B) Ig [IgG + IgM] response induced by 38-BP-Enol vaccination. (C-D) Ig [IgG + IgM] response induced SSU1-BP-SSU2 vaccination. Mice (n =10) were immunized with 2 doses of 38-BP-Enol (6 µg dose) and SSU1-BP-SSU2 (6 µg dose) with Quil-A® adjuvant. Immunogenicity was analyzed by ELISA using either BP or 38-BP-Enol or SSU1-BP-SSU2 as coating. Statistical analysis was performed using unpaired *t* tests Mann–Whitney. Each dot represents a single vaccinated mouse (n = 10 mice per group), with horizontal bars representing the mean of the group. *P* values for each case tested were (A) ***$p < 0.0001$, (B) **$p < 0.0021$, (C) ***$p < 0.0001$ and (D) ***$p < 0.0005$.

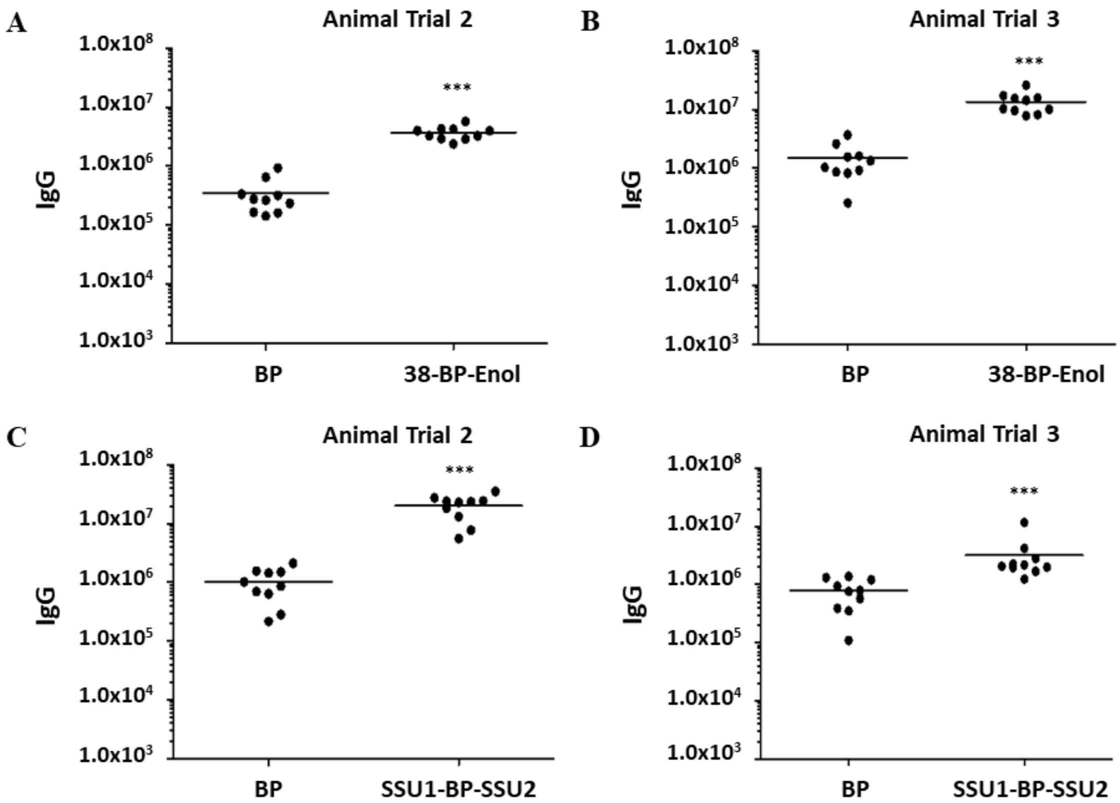

Figure S6. IgG response induced by BP vaccines for the second and third animal trials. (**A**,**B**) IgG response induced by 38-BP-Enol vaccination. (**C**,**D**) IgG response induced SSU1-BP-SSU2 vaccination. Mice ($n = 10$) were immunized with 2 doses of 38-BP-Enol (6 µg dose) and SSU1-BP-SSU2 (6 µg dose) with Quil-A® adjuvant. Immunogenicity was analyzed by ELISA using either BP or 38-BP-Enol or SSU1-BP-SSU2 as coating. Statistical analysis was performed using unpaired t tests Mann–Whitney. *P* values for each case tested were (**A**) ***$p < 0.0001$, (**B**) ***$p < 0.0001$, (**C**) ***$p < 0.0001$ and (**D**) ***$p < 0.0001$. Each dot represents a single vaccinated mouse ($n = 10$ mice per group), with horizontal bars representing the mean of the group.

References

1. Slater, J.; Allen, A.; May, J.; Bolitho, S.; Lindsay, H.; Maskell, D. Mutagenesis of Streptococcus equi and Streptococcus suis by transposon Tn917. *Veterinary microbiology*. **2003**, *93*, 197–206.
2. Amara, A.A.; Rehm, B.H. Replacement of the catalytic nucleophile cysteine-296 by serine in class II polyhydroxyalkanoate synthase from Pseudomonas aeruginosa-mediated synthesis of a new polyester: Identification of catalytic residues. *Biochemical Journal*. **2003**, *374*, 413–421.
3. Rubio Reyes, P.; Parlane, N.A.; Wedlock, D.N.; Rehm, B.H.A. Immunogencity of antigens from *Mycobacterium tuberculosis* self-assembled as particulate vaccines. *Int. J. Med. Microbiol*. **2016**, *306*, 624–632.
4. Peters, V.; Rehm, B.H.A. In vivo monitoring of PHA granule formation using GFP-labeled PHA synthases. *FEMS Microbiol Lett*. **2005**, *248*, 93–100.
